# Antimicrobial Susceptibility Profiles of Commensal *Escherichia coli* Isolates from Turkeys in Hungarian Poultry Farms Between 2022 and 2023

**DOI:** 10.3390/antibiotics14030305

**Published:** 2025-03-16

**Authors:** Ákos Jerzsele, Ádám Kerek, Franciska Barnácz, Bence Csirmaz, Ábel Szabó, László Kovács

**Affiliations:** 1 Department of Pharmacology and Toxicology, University of Veterinary Medicine, István utca 2, H-1078 Budapest, Hungary; jerzsele.akos@univet.hu (Á.J.); barnacz.franciska@student.univet.hu (F.B.); csirmaz.bence@student.univet.hu (B.C.); szabo.abel@student.univet.hu (Á.S.); 2 National Laboratory of Infectious Animal Diseases, Antimicrobial Resistance, Veterinary Public Health and Food Chain Safety, University of Veterinary Medicine, István utca 2, H-1078 Budapest, Hungary; kovacs.laszlo@univet.hu; 3 Department of Animal Hygiene, Herd Health and Mobile Clinic, University of Veterinary Medicine, István utca 2, H-1078 Budapest, Hungary; 4 Poultry-Care Kft., Lehel út 21, H-5052 Újszász, Hungary

**Keywords:** *Escherichia coli*, antimicrobial resistance, minimum inhibitory concentration, MIC, poultry, turkeys, Hungary

## Abstract

**Background**: The global spread of antimicrobial resistance (AMR) has prompted the critical importance of regular monitoring. *Escherichia coli*, a widely distributed facultative anaerobic pathogen, is significant both in terms of the clinical diseases that it causes and as a reservoir of antimicrobial resistance, with notable implications for both animal and public health. Within the poultry industry, the turkey sector is an emerging and internationally significant branch. **Methods**: Our objective was to assess the antimicrobial resistance profile of commensal *Escherichia coli* strains isolated from large-scale turkey flocks in Hungary using minimum inhibitory concentration (MIC) determination. **Results**: A total of 470 isolates were analyzed, revealing that 61.5% of the strains were resistant to amoxicillin, while 18.5% were resistant to amoxicillin–clavulanic acid. The resistance observed against enrofloxacin (62.8%) and ceftriaxone (24%) is concerning. Comparison with human resistance data showed a similar resistance rate for amoxicillin and ampicillin, as well as amoxicillin–clavulanic acid and cephalosporins. However, for other active substances, the situation was significantly worse in veterinary medicine. **Conclusions**: The lower resistance to amoxicillin–clavulanic acid indicates that most strains are β-lactamase producers. Our findings underscore the necessity of regular and comprehensive surveillance, which can establish temporal trends over time. Incorporating data on antibiotic usage into future studies could facilitate the exploration of relevant correlations. Additionally, the next-generation sequencing of multidrug-resistant strains could help elucidate the genetic basis of resistance.

## 1. Introduction

Turkey meat ranks second after broiler chicken meat in global poultry production. In 2016, global demand for turkey meat increased by 9%, resulting in an annual production of 6.2 million tons, valued at USD 13.6 billion. By 2025, this figure is projected to reach 6.7 million tons. The United States leads turkey meat production (2.7 million tons, 43%), consumption (2.519 million tons, 41%), and export (216,000 tons), while Mexico (157,000 tons) and Germany (117,000 tons) dominate imports [1].

Alongside chickens, turkeys are a significant poultry species primarily raised for meat in Hungary, with egg production also important in Canada. Like chickens, turkeys harbor antibiotic-resistant bacteria [2,3]. Turkey meat is globally favored for its taste, low fat content, and high-quality protein. However, rearing turkeys is challenging, especially during the early life stages, due to their high environmental and nutritional demands and susceptibility to infectious diseases, particularly primary and secondary bacterial infections [4,5].

Turkey farming poses unique challenges in managing antimicrobial resistance, as turkeys are typically reared for longer periods and are exposed to higher levels of antibiotics compared to broilers, increasing the risk of resistance development [6,7]. This is further compounded by their susceptibility to bacterial infections, necessitating frequent antibiotic treatments [8]. Commensal *E. coli* from turkeys not only serve as reservoirs for resistance genes but may also act as zoonotic agents, contributing to the dissemination of AMR to humans via foodborne pathways [9]. Therefore, monitoring AMR in turkeys is critical for mitigating both veterinary and public health risks [10].

Annually, Hungary produces 7.5–8.5 million turkeys, yielding 90,000 tons of live weight, from which 65,000–70,000 tons of products are made. Between 30,000 and 35,000 tons are consumed domestically and the rest exported [11].

Antimicrobial resistance (AMR) is one of the most pressing animal and public health concerns worldwide [12,13]. In livestock, AMR monitoring programs began in the Netherlands in 1998 [14]. At the European Union conference held in Copenhagen that same year, the Danish government proposed recommendations emphasizing the precise monitoring of antimicrobial resistance in relation to specific antibiotic treatments and their effects in both animal and public health sectors [15]. The monitoring of antimicrobial resistance and antibiotic usage in animals in the Netherlands (MARAN), established as a result, has since reported regularly on its findings [16]. Between 1998 and 2009, MARAN observed a continuous increase in resistant isolates, including resistance to critically important antimicrobials such as third-generation cephalosporins and fluoroquinolones, as well as a notable rise in multidrug-resistant isolates in poultry, swine, and calves [17,18,19,20].

*Escherichia coli* (*E. coli*), a Gram-negative, rod-shaped bacterium belonging to the Enterobacteriaceae family, is a facultative anaerobic pathogen. Most *E. coli* strains colonizing the intestines of chickens are commensals and part of the normal gut microbiome. However, some strains of *E. coli,* classified under the extraintestinal pathogenic *E. coli* (ExPEC) category, are avian pathogenic (APEC) [21,22,23,24]. APEC strains can cause colibacillosis, which is a leading cause of increased mortality, high treatment costs, and carcass condemnations in the poultry industry globally [25]. The contamination of chicken meat with *E. coli* primarily occurs during evisceration at slaughterhouses, posing a significant public health risk due to the transmission of antimicrobial resistance genes and the potential for pathogenic shiga toxin-producing *E. coli* (STEC) strains [9,26,27].

*E. coli* strains can be categorized into four main phylogenetic groups (A, B1, B2, and D) [28,29]. Groups A and B1 are often associated with multidrug-resistant strains that may contribute to the transmission of AMR between animals, the environment, and humans [30]. However, some studies have failed to establish a definitive correlation between phylogenetic groups and multidrug resistance [31,32]. Isolates from both humans and poultry have been identified in groups A and B1, whereas groups B2 and D are primarily isolated as extraintestinal pathogens in humans and birds [33,34]. A comprehensive slaughterhouse study found that 66.7% of isolates from turkey meat belonged to group A, 16.7% to group B1, and 16.7% to group D [35].

A study analyzing resistance rates of *E. coli* isolates from animals slaughtered between 2009 and 2012 revealed that isolates from turkeys exhibited higher resistance levels compared to other animals. The study concluded that resistance development is a multifactorial phenomenon not solely dependent on antibiotic usage [36]. Another study in 2016 found that resistance frequencies in turkeys were comparable to those in broiler chickens [37]. However, due to their longer rearing period and more intensive care requirements, turkeys present a higher risk for farmworkers to acquire resistant bacteria [6]. Reducing the proportion of resistant strains induced by selective antibiotic pressure can be achieved by minimizing antibiotic usage and employing alternatives [38] such as propolis [7,8], essential oils [10], probiotics [39], medium-chain fatty acids [40], and plant extracts [41,42]. Antimicrobial peptides are also gaining wider application [43]. Prudent and reduced antibiotic use is particularly critical in the swine industry, the largest antibiotic user [44], and the poultry industry, the second-largest user [45]. Additionally, selecting active substances must always be based on appropriate pharmacokinetic/pharmacodynamic studies [46].

Given its economic significance and prominent export market, we aimed to assess the nationwide antimicrobial resistance status in turkeys through comprehensive surveys.

## 2. Results

### 2.1. Regional Distribution and Origin of Received Samples

**Figure 1:**
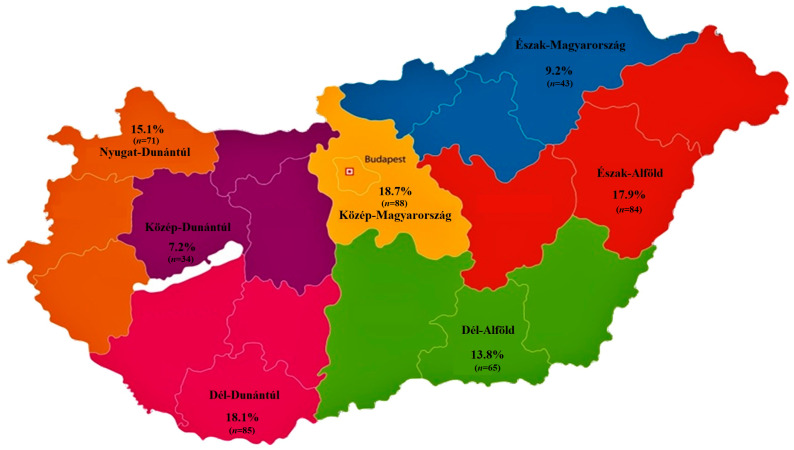
The regional distribution of *Escherichia coli* strains (*n* = 470) isolated from turkeys across the seven administrative regions of Hungary.

We analyzed a total of 470 commensal *E. coli* isolates obtained from large-scale turkey farms. Regarding the regional distribution (Figure 1), 9.2% of the samples originated from the Észak-Magyarország region, 7.2% from Közép-Dunántúl, 18.7% from Közép-Magyarország, 15.1% from Nyugat-Dunántúl, 13.8% from Dél-Alföld, 18.1% from Dél-Dunántúl, and 17.9% from the Észak-Alföld. Among the samples, 52.8% were cloacal swabs, while 47.2% were respiratory swabs. The isolates were derived from meat-producing flocks (71.9%) and breeder flocks (28.1%), with age groups corresponding to juvenile and adult birds based on the production purpose. A majority of the samples (87.2%) were collected from smaller farms (5001–50,000), and only 12.8% originated from medium-sized farms (50,001–100,000).

### 2.2. Antimicrobial Susceptibility Testing

We examined whether resistance levels for each antimicrobial agent differed significantly based on various grouping criteria for the isolates (Table 1). The results indicate that the sampling source (respiratory or cloacal) had no effect on resistance levels. This can likely be attributed to the birds’ behavior of pecking in fecally contaminated environments. The most influential factor was the age group. Significant differences in resistance were observed for five antimicrobials: ceftriaxone, imipenem, florfenicol, spectinomycin, and colistin dependent on age. It should be noted that production type correlated perfectly with age group (juvenile vs. adult), as all meat birds were juveniles, while breeder flocks consisted of adult birds. The size of the flock was another influential factor, with significant differences noted for imipenem, florfenicol, enrofloxacin, and colistin resistance between small (5001–50,000) and medium-sized (50,001–100,000) farms.

Minimum inhibitory concentrations (MICs) were determined for 15 antimicrobial agents, with clinical breakpoints available for 11 of them, and a frequency table was prepared (Table 2). Frequency data for agents without clinical breakpoints are presented in Supplementary Table S1.

MIC_50_ and MIC_90_ values were calculated for each antimicrobial. MIC_50_ represents the minimum concentration required to inhibit 50% of the tested bacterial population, while MIC_90_ is the concentration required to inhibit 90%. These metrics are crucial in evaluating antibiotic efficacy against specific bacterial populations and inform clinical decision making by guiding the selection of the most effective treatment. In our study, MIC_50_ values were below the clinical breakpoints for amoxicillin–clavulanate, ceftriaxone, imipenem, spectinomycin, and colistin.

Epidemiological cut-off values (ECOFF) values, as defined by the European Committee on Antimicrobial Susceptibility Testing (EUCAST) [47,48], were also included in the table. ECOFF differentiates between wild-type populations and strains altered by mutations or resistance mechanisms. It represents the highest MIC value observed in wild-type strains of a microorganism. Using ECOFF values in clinical practice and public health monitoring offers significant advantages. In this study, MIC_50_ values for ceftriaxone, imipenem, and colistin were below the corresponding ECOFF thresholds, indicating these populations largely remained within the wild-type sensitivity range.

The proportion of multidrug-resistant strains was determined, with strains exhibiting resistance to at least three antibiotics accounting for 26.7% of all isolates.

**Figure 2:**
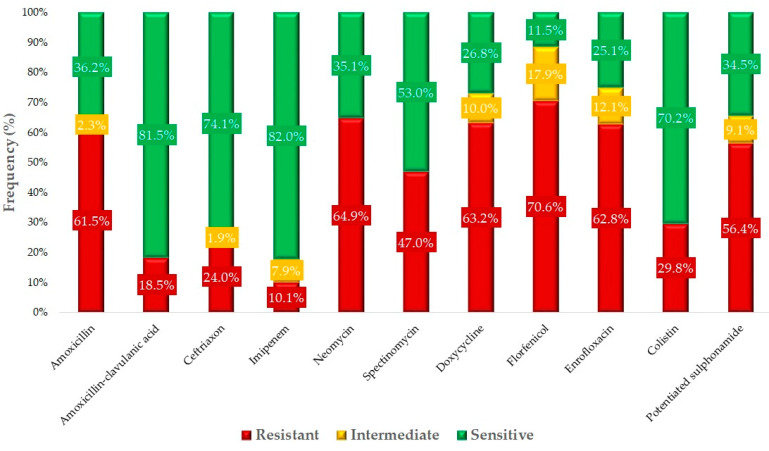
Susceptibility profile of *Escherichia coli* samples isolated from turkeys (*n* = 470) to active substances of animal and public health importance.

Based on clinical breakpoints, the susceptibility profiles for each antimicrobial agent were determined (Figure 2). The highest resistance was observed for florfenicol (70.6%), followed by doxycycline (63.2%). The resistance rates of 61.5% for amoxicillin and 18.5% for amoxicillin–clavulanate suggest that a significant portion of the isolates are phenotypically β-lactamase producers. Alarmingly, resistance to enrofloxacin, a critically important antimicrobial agent, was 62.8% in the turkey sector.

**Figure 3:**
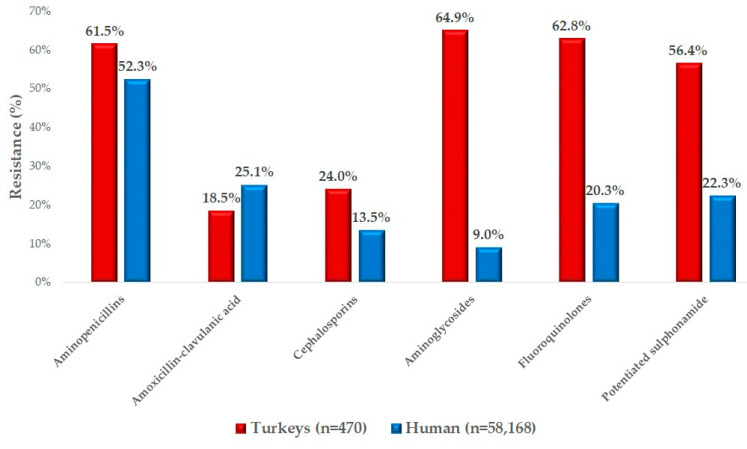
A comparison of commensal *Escherichia coli* strains isolated from turkeys and available human resistance data.

We compared our findings with human antimicrobial resistance data for Hungary (Figure 3). For amoxicillin, turkey isolates exhibited a 61.5% resistance rate, slightly higher than the 52.3% resistance observed for ampicillin in human healthcare, with both antibiotics belonging to the aminopenicillin class used for comparison. Conversely, resistance to amoxicillin–clavulanate was lower in turkey isolates (18.5%) compared to the 25.1% observed in human healthcare. For all other antimicrobials compared, resistance rates were consistently higher in veterinary settings in the case of *E. coli* bacteria. Particularly concerning is the 62.8% resistance to enrofloxacin observed in turkey isolates, likely reflecting the extensive use of fluoroquinolones in the poultry sector.

**Figure 4:**
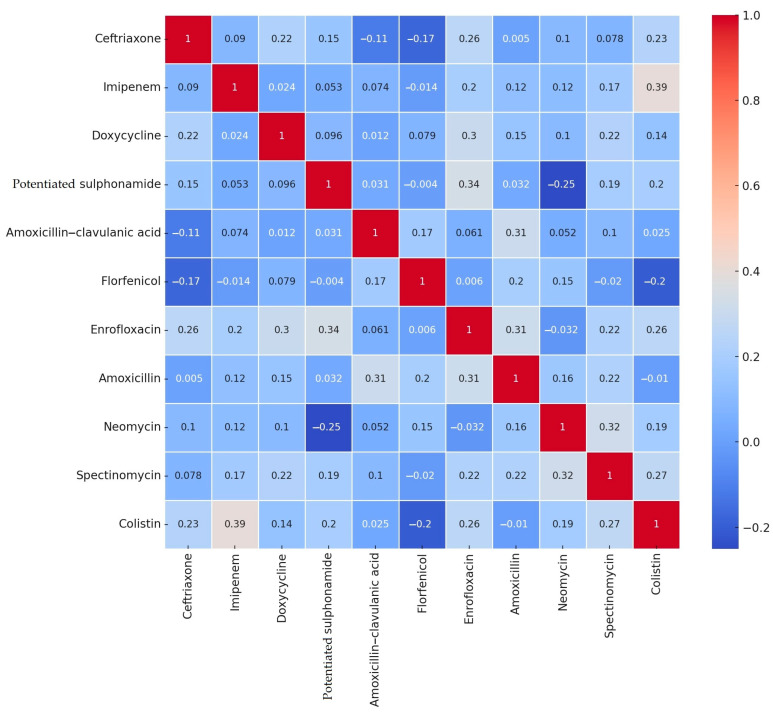
Correlation analysis of antimicrobial resistance rates of *Escherichia coli* strains isolated from turkeys (*n* = 470) by drug substance.

Figure 4 presents a correlation matrix illustrating the relationships between resistance levels to various antimicrobials. Correlation analysis is crucial in antimicrobial resistance studies as it helps identify co-resistance patterns, which can provide insight into potential genetic linkages or co-selection mechanisms driven by antimicrobial use.

Our findings indicate that the strongest positive correlation was observed between imipenem and colistin (0.39), as well as amoxicillin and amoxicillin–clavulanic acid (0.31). This suggests that isolates resistant to one of these critically important last-resort antibiotics are more likely to exhibit resistance to the other as well. Such co-resistance patterns may be associated with shared resistance mechanisms, such as the presence of mobile genetic elements carrying multiple resistance genes.

The most negative correlation was found between neomycin and potentiated sulfonamide (−0.25), indicating that resistance to these two antimicrobial classes may be inversely related. This could be due to differences in the selection pressures exerted by these antibiotics or potential fitness costs associated with specific resistance mechanisms.

Additionally, some neutral relationships were identified, such as florfenicol and potentiated sulfonamide (−0.004) and florfenicol and enrofloxacin (0.006). These minimal correlation values suggest that resistance to these antibiotics occurs independently, without a strong tendency for co-selection.

**Figure 5:**
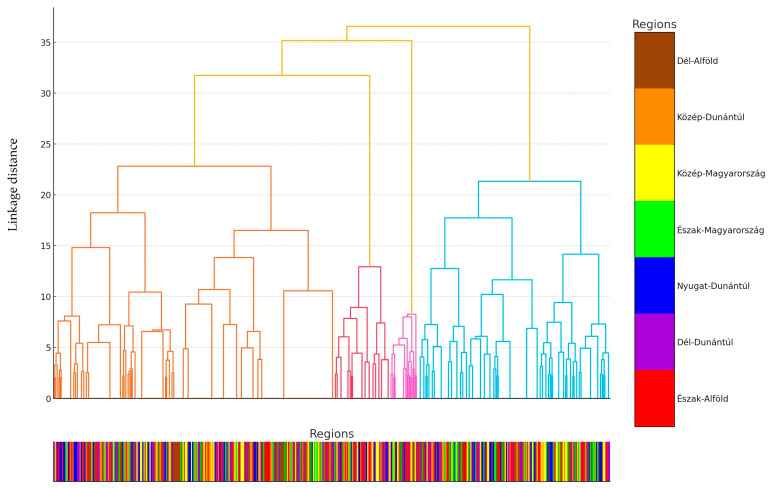
Cluster analysis of *Escherichia coli* strains isolated from turkeys (*n* = 470), the classification of samples by region, antimicrobial resistance, and color coding are shown below the horizontal axis. Different colors are shown in the figure to facilitate separation of the main clusters. The colored bar at the bottom of the dendrogram represents the regional origin of each isolate, corresponding to the color scheme in the legend. Each vertical segment in this bar denotes an individual isolate, and its color indicates the administrative region from which it was collected. This allows for a quick visual assessment of how isolates from different regions cluster together or remain dispersed across multiple clusters.

Cluster analysis was performed for each sample (Figure 5). To enhance clarity, instead of labeling samples by their individual identification numbers, they were categorized based on their respective regions of origin. These regions were color-coded, and a line indicating the corresponding region’s color was added to the explanatory section of the horizontal axis in the graph.

Isolates from Dél-Alföld (brown) and Közép-Dunántúl (orange) tend to cluster together, suggesting that antimicrobial resistance patterns in these regions may be influenced by similar antibiotic usage practices, farm management strategies, or environmental conditions. The clustering pattern in these regions indicates a relatively homogenous resistance profile, possibly due to localized selective pressures or the presence of common resistance determinants.

In contrast, isolates from Nyugat-Dunántúl (blue) and Észak-Magyarország (green) are more widely dispersed across multiple clusters, indicating greater variability in resistance profiles within these regions. This suggests that farms in these regions may differ significantly in terms of antibiotic usage, biosecurity measures, or exposure to external resistance sources, resulting in more diverse bacterial populations.

Some clusters contain isolates from multiple regions, indicating that certain resistance determinants are not confined to a single geographic area. This may suggest the role of animal trade, feed contamination, shared farming practices, or environmental reservoirs in the dissemination of antimicrobial resistance genes across regions.

**Figure 6:**
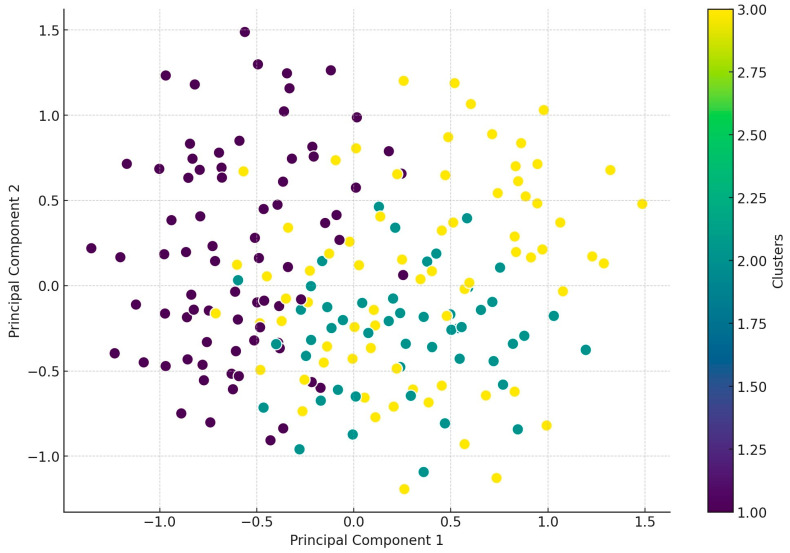
Principal component analysis (PCA) of the samples according to the distribution of the samples in three main clusters after cluster analysis-based antimicrobial resistance. Samples classified as Cluster 1 are shown in purple, Cluster 2 in green, and Cluster 3 in yellow.

Subsequently, we conducted a principal component analysis (PCA) to explore patterns and relationships within the dataset and to reduce the dimensionality of high-dimensional data while preserving the majority of the data’s variance (Figure 6). The regional distribution of the clusters was generally balanced, with some notable deviations: Cluster 1 had a significantly higher number of samples from the Dél-Alföld region, Cluster 2 had fewer samples than average from the Közép-Magyarország region, and Cluster 3 included a higher-than-average number of samples from the Dél-Dunántúl region.

In the PCA plot, the distribution of clusters indicates certain trends within the dataset. Cluster 1 (dark purple) appears to be relatively well separated from the others, particularly along the first principal component. Cluster 2 (green) shows some degree of overlap with both Cluster 1 and Cluster 3, suggesting partial similarity in resistance patterns. In contrast, Cluster 3 (yellow) exhibits a more scattered distribution without a distinct grouping, which may indicate higher within-cluster variability. This dispersion suggests that the factors contributing to the classification of Cluster 3 are more heterogeneous or less dominant along the first two principal components. Further analysis may be needed to determine whether additional dimensions capture more distinct clustering patterns for this group. In Cluster 1, represented in purple, high resistance levels were observed against florfenicol, potentiated sulfonamide, and doxycycline. In Cluster 2, represented in yellow, high resistance was detected against imipenem, enrofloxacin, amoxicillin, and potentiated sulfonamide. In Cluster 3, represented in green, high resistance was observed against potentiated sulfonamide, doxycycline, enrofloxacin, amoxicillin, and neomycin.

## 3. Discussion

We conducted a nationwide susceptibility study on *E. coli* commensal strains isolated from large-scale turkey farms (*n* = 470) across seven regions of Hungary, determining their minimum inhibitory concentrations (MICs).

Our findings revealed a 61.5% resistance rate to amoxicillin. In comparison, Shrestha et al. reported a lower resistance rate of 31.2% [49], while Suwono et al. observed a similar 67.6% resistance rate to ampicillin, which holds higher relevance in human healthcare [50]. Agunos et al. documented an increase in resistance from 50% to 70% between 2013 and 2017 [51]. These findings align with our results and underscore the widespread and long-term use of amoxicillin in poultry farming. Discrepancies in resistance levels may reflect regional differences in antibiotic usage practices.

*E. coli* strains frequently produce β-lactamase enzymes, which degrade penicillin enzymatically. Although clavulanic acid is not authorized for use in poultry, investigating its combination with amoxicillin holds significant importance for both veterinary and public health. We observed an 18.5% resistance rate to amoxicillin–clavulanate, whereas Boulianne et al. reported a higher resistance rate of 34.5% [52]. Our findings, compared to those for amoxicillin alone, support the conclusion that a substantial proportion of isolates produce β-lactamase enzymes. Differences in phylogenetic backgrounds and varying selection pressures may explain the lower resistance rates observed in other regions.

The use of cephalosporins is similarly not authorized in poultry; however, the global increase in resistance against these antibiotics is concerning. We observed a 24% resistance rate to ceftriaxone, while Cook et al. reported no resistant strains [2], Boulianne et al. noted a higher rate of 33.1% [52], and Agunos et al. observed resistance levels ranging from 1% to 10% between 2013 and 2017 [51]. Although these drugs are not used in poultry, resistance to them poses a significant public health concern. This resistance is likely driven by selection pressure from other antimicrobials and the co-resistance mechanisms shared between certain antibiotics.

We detected a 63.2% resistance rate to doxycycline, aligning with findings by Shrestha et al. (61.7%) [49] and Grobbel et al. (49%) [53]. The extensive, decades-long use of tetracyclines, coupled with their poor oral absorption, has likely contributed to the high resistance rates observed.

Among aminoglycosides, neomycin showed a resistance rate of 64.9% in our study. Shrestha et al.’s combined analysis (neomycin, gentamicin, streptomycin) reported a resistance rate of 45% [49], Boulianne et al. observed rates of 61.1% for aminoglycosides overall, and 27.9% for neomycin specifically [52]. For gentamicin, Grobbel et al. reported 5% resistance [53], and Suwono et al. found a rate of 11.8% [50]. Agunos et al. documented a steady increase in aminoglycoside resistance from 10% to 30% between 2013 and 2017 [51]. Aminoglycosides remain critical in human medicine due to their broad spectrum of activity. However, the potential for unidirectional cross-resistance within the group and the high resistance rates in veterinary contexts have gradually diminished their utility.

We observed a 62.8% resistance rate to enrofloxacin, which is particularly concerning as it is a critically important antibiotic whose usage must be significantly restricted in the future. The poultry industry remains one of the largest consumers of fluoroquinolones. In contrast, studies conducted by Boulianne et al. [52] and Cook et al. [2] reported no resistance to enrofloxacin or ciprofloxacin, while Grobbel et al. found 35% ciprofloxacin resistance [53], and Suwono et al. reported 28% [50]. These differences likely reflect variations in antibiotic usage practices across nations. Current regulations mandate that enrofloxacin use in veterinary medicine is limited to cases with confirmed sensitivity and only as a second-line treatment. Ciprofloxacin monitoring is increasingly emphasized due to the metabolism of enrofloxacin into ciprofloxacin, a compound of critical importance in human medicine.

The high resistance rates observed for fluoroquinolones and florfenicol underscore the urgent need for alternative therapeutic strategies. Natural alternatives, such as essential oils and plant extracts, have shown promise in reducing bacterial infections in poultry while minimizing selective pressure for resistance [10,54,55]. Additionally, targeted surveillance incorporating antibiotic usage data could provide clearer insights into the drivers of resistance [51].

For potentiated sulfonamides (trimethoprim and sulfamethoxazole at a 1:19 ratio), we observed a 56.4% resistance rate. Shrestha et al. reported a lower rate of 30.4% [49], while Boulianne et al. documented a much higher rate of 82.7% [52]. The widespread and long-term use of this antibiotic class explains the high resistance levels reported in numerous studies, although reduced use in recent years has shown signs of improving sensitivity.

Resistance to colistin was observed at 29.8%, compared to 3.2% in turkey samples reported by Nobili et al. [56], and no resistance in German turkey farms in 2014 by Mesa-Varona et al., which rose to 9.5% in 2017 [57]. These values are alarming, and further investigation using next-generation sequencing is essential to uncover underlying causes. The prolonged rearing period of turkeys may allow for more frequent and extended antibiotic treatments, contributing to higher resistance levels.

Florfenicol resistance was recorded at 70.6%. Hu et al. found all isolates from poultry farm environments to be resistant [58], whereas Gambi et al. observed no resistant strains in laying hens [59]. The high resistance levels noted in our study are concerning given the broad-spectrum nature of florfenicol. The discrepancies in the literature suggest substantial differences in its usage across countries.

Spectinomycin showed a resistance rate of 47.0%, compared to 4.7% in turkey meat samples reported by Piccirillo et al. [60]. This result aligns with the high resistance rates observed for neomycin, as there is a significant potential for cross-resistance within this antibiotic group.

For imipenem, we observed a 10.2% resistance rate, while Moawad et al. reported no resistant strains [61]. As a last-resort antibiotic reserved for human medicine, any level of resistance is unacceptable. It is worth noting that imipenem is known to have stability issues, which might affect the reliability of these findings. Future studies should focus on more stable carbapenems, such as meropenem or ertapenem, and confirm these results using next-generation sequencing.

Our findings revealed no significant differences in resistance profiles between respiratory and cloacal sample sources. However, the production type (meat vs. breeding) was a determining factor, likely due to differences in rearing duration and antibiotic use. Similar results were observed in our previous studies on chickens, where production type influenced resistance profiles [62]. This underscores the need for future resistance monitoring to be stratified by age groups.

When comparing our results with human resistance data, a similar pattern was noted for amoxicillin-ampicillin and amoxicillin–clavulanic acid. Carmona-Cartaya et al. reported a 68.3% resistance rate to ampicillin in human uropathogenic strains [63], closely aligning with the 61.5% resistance in turkeys and 52.3% in humans observed in this study. For cephalosporins, resistance levels were markedly higher in veterinary isolates compared to human data. The greatest disparity was noted for aminoglycosides, where veterinary resistance was sevenfold higher, while fluoroquinolones and potentiated sulfonamides showed threefold greater resistance in veterinary isolates. Carmona-Cartaya et al. documented 54.8% resistance to ciprofloxacin in human uropathogenic strains [63], which is comparable to the 62.8% resistance that we observed in turkeys but significantly higher than the 20.3% resistance reported in human data. Similarly, the study reported 49.5% resistance to potentiated sulfonamides in humans [63], close to our finding of 56.4% in turkeys but substantially exceeding the 22.3% resistance observed in human cases. These compounds are particularly significant as amoxicillin is a first-line treatment for *E. coli* urinary tract infections in veterinary medicine [64], while fluoroquinolones and potentiated sulfonamides are primary options for treating prostatitis and other related conditions in both human and veterinary contexts [65,66].

Regional variations in resistance rates may reflect differences in biosecurity practices and farm-level antibiotic policies [67,68]. Understanding these differences through targeted interventions could inform more effective AMR management strategies [52].

In Hungary, antibiotic use in the poultry sector is strictly regulated in accordance with European Union directives. The prophylactic use of antibiotics is prohibited; they may only be administered for therapeutic purposes based on a veterinary diagnosis and under strict veterinary supervision. The regulatory framework aims to minimize antimicrobial resistance while ensuring animal health and welfare [69]. The system of animal welfare subsidies incentivizes farmers to reduce antibiotic use and adopt alternative measures. These subsidies cover additional costs associated with alternative methods and compensate for potential revenue losses, thereby promoting reduced antibiotic dependence and improving the overall health and welfare of poultry flocks.

Moreover, the continuous monitoring of antimicrobial resistance trends in the Hungarian poultry sector informs guidelines for antibiotic use. Farmers and veterinarians are encouraged to implement preventive measures and alternatives to reduce the necessity of antibiotics while maintaining flock health. These combined efforts ensure the responsible use of antibiotics in the Hungarian poultry industry, mitigating the risk of antimicrobial resistance development while safeguarding animal health and productivity.

## 4. Materials and Methods

### 4.1. The Origin of Strains and Human Data

In Hungary, veterinarians routinely collect samples from healthy live animals as part of diagnostic procedures The examined strains were collected between 2022 and 2023 as part of routine diagnostic investigations serving large-scale livestock farms. The selection of farms was entirely random, with the only criterion being that samples were collected from at least three farms per administrative region. The animals selected for sampling were chosen entirely at random. The selection criteria prioritized geographical coverage, along with voluntary participation, ensuring representation from all regions of Hungary. Cloacal and respiratory sample types were preferred because *E. coli* is a natural component of the gut microbiota and can also colonize the oropharyngeal cavity due to natural pecking behavior.

The samples included detailed information on the source organ (trachea or cloaca), the originating municipality, the production purpose of the flock (meat or breeding), the corresponding age group (juvenile or adult), and the flock size category (5001–50,000; 50,001–100,000). Based on the municipalities, the samples were classified into Hungary’s seven administrative regions.

Strains isolated from these diagnostic samples were cultured on ChromoBio^®^ Coliform medium (Biolab Zrt., Budapest, Hungary), which supports robust growth and produces blue-colored colonies indicative of *E. coli*. The blue colonies were then subcultured onto tryptone soy agar (Biolab Zrt., Budapest, Hungary) to establish pure colony cultures. Pure cultures from the samples were stored at −80 °C using the Microbank™ system (Pro-Lab Diagnostics, Richmond Hill, ON, Canada).

Human resistance data were provided by the Hungarian National Centre for Public Health and Pharmacy. The human resistance data referred to ampicillin, while, in veterinary cases, amoxicillin was considered. For third-generation cephalosporins, the comparison included ceftriaxone. Aminoglycoside resistance data were aggregated for gentamicin, tobramycin, and amikacin, with additional specific data for neomycin. Similarly, fluoroquinolone resistance was analyzed as a group, with enrofloxacin examined separately in veterinary cases. Human resistance data, both aggregated and region-specific, were provided in an Excel file with authorization from the National Chief Medical Officer. The dataset included resistance rate percentages.

### 4.2. Minimum Inhibitory Concentration (MIC) Determination

Phenotypic resistance expression was assessed by determining the minimum inhibitory concentration (MIC) values following the methodology of the Clinical Laboratory Standards Institute standard M07 (CLSI, 2018) [70]. Breakpoints were also established based on CLSI guidelines [70] and compared with the ECOFF defined by the EUCAST. Most clinical breakpoints were based on CLSI standards; however, for certain agents lacking CLSI-defined breakpoints, the literature references were used, including amoxicillin–clavulanate [52], neomycin [71], spectinomycin [52], and colistin [72].

The bacterial strains stored at −80 °C were suspended in 3 mL of cation-adjusted Müller Hinton broth (CAMHB) one day before testing and incubated at 37 °C for 18–24 h. The experiments were conducted using 96-well microtiter plates (VWR International, LLC, Debrecen, Hungary). All wells, except for those in the first column, were filled with 90 µL of CAMHB. Stock solutions of the tested agents (Merck KGaA, Darmstadt, Germany) were prepared at a concentration of 1024 µg/mL following CLSI guidelines [70]. From this, a 512 µg/mL working solution was prepared by diluting it 1:1 with broth, and 180 µL of this solution was added to the first column of the plate. A twofold serial dilution was then performed across the plate. Excess 90 µL from the 10th column was discarded, leaving 90 µL in each well. Bacterial suspensions were adjusted to 0.5 McFarland standard using a nephelometer (ThermoFisher Scientific, Budapest, Hungary) and inoculated into the wells starting from the 11th column in reverse order, at 10 µL per well [70]. The results were evaluated using the Sensititre™ SWIN™ automatic MIC reader (ThermoFisher Scientific, Budapest, Hungary) and VIZION system software version 3.4 (ThermoFisher Scientific, Budapest, Hungary, 2024). The reference isolated used was *Escherichia coli* (ATCC 25922).

### 4.3. Statistical Analyses

Statistical analysis of the data was performed using R program version 4.1.0 [73]. Normality was assessed using the Shapiro–Wilk test, and non-parametric tests were applied for data that did not follow a normal distribution. Differences in resistance levels to individual agents across various groups were analyzed using the Kruskal–Wallis test [74], a non-parametric method suitable for comparing medians across multiple groups. This approach is ideal for evaluating differences among diverse categories. Post hoc tests were subsequently performed to identify specific group-level associations. Pairwise comparisons were conducted using the Mann–Whitney U test [75] and *t*-tests, with Bonferroni correction applied to adjust for inflated *p*-values resulting from multiple comparisons [76]. While the Bonferroni correction reduces the likelihood of Type I errors, it increases the risk of Type II errors (failure to detect true differences). Additional correlation analyses were performed to explore relationships between individual agents. A correlation coefficient of +1 indicates a perfect positive correlation, meaning that, as one variable increases, the other also increases. A value of 0 indicates no correlation, implying no relationship between the variables, while a value of −1 signifies a perfect negative correlation, where an increase in one variable corresponds to a decrease in the other. Positive correlations signify that both variables either increase or decrease together, while negative correlations indicate an inverse relationship. PCA [64] was utilized to identify patterns and similarities among samples, followed by hierarchical cluster analysis, which was visualized in a dendrogram [77]. The dendrogram provides a visual representation of inter-sample distances and clustering hierarchies.

## 5. Conclusions

In conclusion, our findings align with the international literature in several aspects. The high levels of resistance observed in the poultry sector can be attributed not only to decades of antibiotic use but also to the increasing intensification of the industry. As humanity’s demand for animal-derived protein grows, so does the need for enhanced productivity and larger-scale farms, which increase both biosecurity challenges and associated risks. The widespread resistance to critically important antibiotics is particularly concerning. Commensal strains may act as reservoirs for resistance, facilitating the dissemination of resistance genes, especially in the case of *E. coli*. Our results highlight the necessity of regular, comprehensive surveillance to monitor resistance trends over time. By integrating antibiotic usage data with human resistance information, a stronger connection can be established between veterinary and public health under the One Health approach. For multidrug-resistant strains, genetic analyses can identify the specific genes driving resistance, paving the way for more targeted interventions.

To address the high resistance rates observed, it is essential to implement stricter antibiotic stewardship programs—including improving the on-farm biosecurity—and explore the use of alternative therapeutic options. Veterinarians should promote preventive measures such as vaccination programs, probiotic supplementation, and improved farm hygiene to reduce infection risks and lower antibiotic dependence. Policymakers should enforce stricter antibiotic stewardship programs, including regulations on critically important antibiotics, mandatory resistance monitoring, and stricter biosecurity protocols. Poultry producers should be provided with education programs focusing on responsible antibiotic use, alternative treatment strategies, and farm-level biosecurity improvements to minimize resistance emergence.

Future research should focus on integrating genomic analyses of multidrug-resistant strains to identify resistance mechanisms and guide intervention strategies. Collaboration between veterinary and human health sectors is critical for establishing unified policies to combat AMR.

## Figures and Tables

**Table 1:** Statistical analysis of each aspect of resistance.

Antibiotics	Respiratory–Cloaca	Meat–Breeding	^3^ Young–^4^ Adult	^5^ Small–^6^ Medium
	*p*-Values			
Ceftriaxone	1.0000	0.0015 *	0.0015 *	0.7282
Imipenem	0.7206	0.0023 *	0.0023 *	0.0347 *
Doxycycline	0.1669	0.6840	0.6840	0.1207
^1^ Potentiated sulfonamide	1.0000	0.3791	0.3791	0.2935
^2^ Amoxicillin–clavulanic acid	0.5670	1.0000	1.0000	1.0000
Florfenicol	1.0000	0.0001 *	0.0001 *	0.0002 *
Enrofloxacin	1.0000	0.3556	0.3556	0.0002 *
Amoxicillin	0.3418	0.1059	0.1059	0.8632
Neomycin	0.9131	0.1853	0.1853	0.8703
Spectinomycin	0.8368	<0.0001 *	<0.0001 *	0.4525
Colistin	0.2777	<0.0001 *	<0.0001 *	0.0006 *

* significant difference (*p* < 0.05); ^1^ trimethoprim–sulfamethoxazole 1:19 ratio; ^2^ 1:2 ratio; ^3^ younger than 6 weeks; ^4^ older than 6 weeks; ^5^ small—5001–50,000 birds; and ^6^ medium—50,001–100,000 birds.

**Table 2:** Frequency table of the minimum inhibitory concentration (MIC) values of active substances with breakpoints obtained from *Escherichia coli* samples of turkey origin (*n* = 470). The top row of each agent shows the number of pieces, and the bottom row shows the percentage of each. The vertical red line indicates the breakpoint.

Antibiotic	^1^ BP *	0.001	0.002	0.004	0.008	0.016	0.03	0.06	0.125	0.25	0.5	1	2	4	8	16	32	64	128	256	512	1024	MIC_50_	MIC_90_	^2^ ECOFF
	µg/mL																						µg/mL		
Enrofloxacin	^1^ 2	6	0	1	5	24	24	22	13	13	22	45	35	14	38	55	73	32	22	13	9	4	8	128	0.125
		1.3%	0.0%	0.2%	1.1%	5.1%	5.1%	4.7%	2.8%	2.8%	4.7%	9.6%	7.4%	3.0%	8.1%	11.7%	15.5%	6.8%	4.7%	2.8%	1.9%	0.9%			
Colistin	2	2	0	0	0	11	17	36	45	103	83	33	10	12	6	4	3	1	1	5	21	77	0.5	1024	2
		0.4%	0.0%	0.0%	0.0%	2.3%	3.6%	7.7%	9.6%	21.9%	17.7%	7.0%	2.1%	2.6%	1.3%	0.9%	0.6%	0.2%	0.2%	1.1%	4.5%	16.4%			
Ceftriaxone	^1^ 4			1	3	22	76	135	62	12	12	25	9	10	13	8	4	3	10	19	14	32	0.063	256	0.125
				0.2%	0.6%	4.7%	16.2%	28.7%	13.2%	2.6%	2.6%	5.3%	1.9%	2.1%	2.8%	1.7%	0.9%	0.6%	2.1%	4.0%	3.0%	6.8%			
Imipenem	^1^ 4				2	5	5	27	77	97	110	62	37	30	13	5							0.25	4	0.5
					0.4%	1.1%	1.1%	5.7%	16.4%	20.6%	23.4%	13.2%	7.9%	6.4%	2.8%	1.1%									
^3^ Potentiated sulphonamide	^1^ 4									1	5	11	59	67	40	22	21	15	7	20	42	160	4	64	0.5
										0.2%	1.1%	2.3%	12.6%	14.3%	8.5%	4.7%	4.5%	3.2%	1.5%	4.3%	8.9%	34.0%			
Doxycycline	^1^ 16								4	1	0	29	60	32	47	73	101	97	20	2	3	1	16	64	8
									0.9%	0.2%	0.0%	6.2%	12.8%	6.8%	10.0%	15.5%	21.5%	20.6%	4.3%	0.4%	0.6%	0.2%			
Florfenicol	^1^ 16											2	9	43	84	126	56	28	16	62	38	6	16	256	16
												0.4%	1.9%	9.1%	17.9%	26.8%	11.9%	6.0%	3.4%	13.2%	8.1%	1.3%			
Neomycin	32											8	56	66	13	22	37	129	59	26	37	17	64	512	8
												1.7%	11.9%	14.0%	2.8%	4.7%	7.9%	27.4%	12.6%	5.5%	7.9%	3.6%			
Amoxicillin	^1^ 32						1	2	4	4	17	6	22	73	41	11	16	11	29	29	52	152	128	1024	8
							0.2%	0.4%	0.9%	0.9%	3.6%	1.3%	4.7%	15.5%	8.7%	2.3%	3.4%	2.3%	6.2%	6.2%	11.1%	32.3%			
^4^ Amoxicillin–clavulanic acid	32								4	13	6	15	28	71	138	108	50	23	12	2			8	32	8
									0.9%	2.8%	1.3%	3.2%	6.0%	15.1%	29.4%	23.0%	10.6%	4.9%	2.6%	0.4%					
Spectinomycin	128													1	8	66	64	110	84	68	33	36	64	512	64
														0.2%	1.7%	14.0%	13.6%	23.4%	17.9%	14.5%	7.0%	7.7%			

* BP–breakpoint; ^1^ Clinical Laboratory Standard Institute (CLSI); ^2^ Epidemiological cut-off value (EUCAST); ^3^ 2:1 ratio; and ^4^ trimetophrime–sulphamethoxazole 1:19 ratio.

## Data Availability

The data presented in this study are available from the corresponding author upon reasonable request.

## Supplementary Materials

The following supporting information can be downloaded at https://www.mdpi.com/article/10.3390/antibiotics14030305/s1, Table S1: Frequency table of the minimum inhibitory concentration (MIC) values (µg/mL) for agents without breakpoints in *Escherichia coli* samples derived from turkeys (*n* = 470).
