# Supplementary material for: Antimicrobial Susceptibility Profiles of Commensal Escherichia coli Isolates from Turkeys in Hungarian Poultry Farms Between 2022 and 2023
Source: Antibiotics (Basel). 2025 Mar 16;14(3):305. doi: 10.3390/antibiotics14030305 (PMC11939659; doi:10.3390/antibiotics14030305)
Supplement: Supplementary file 1 [file antibiotics-14-00305-s001.zip › antibiotics-3507526-supplementary.pdf]

Supplementary Table S1 Frequency table of the minimum inhibitory concentration (MIC) values (µg/mL) for agents without breakpoints in *Escherichia coli* samples derived from turkeys (*n*=470). The top row for each agent shows the count, while the bottom row shows the percentage.

Antibiotic	0.001	0.002	0.004	0.008	0.016	0.03	0.06	0.125	0.25	0.5	1	2	4	8	16	32	64	128	256	512	1024	MIC_{50}	MIC_{90}
																						µg/mL	
**Tilozin**											12	6	0	1	1	0	3	2	90	178	177	512	1024
											2.6%	1.3%	0.0%	0.2%	0.2%	0.0%	0.6%	0.4%	19.1%	37.9%	37.7%		
**Tiamulin**										1	2	0	0	2	2	18	47	134	145	58	61	256	1024
										0.2%	0.4%	0.0%	0.0%	0.4%	0.4%	3.8%	10.0%	28.5%	30.9%	12.3%	13.0%		
**Lincomycin**											1	1	0	0	4	2	11	4	23	120	304	1024	1024
											0.2%	0.2%	0.0%	0.0%	0.9%	0.4%	2.3%	0.9%	4.9%	25.5%	64.7%		
**Vancomycin**																	37	19	232	131	51	256	1024
																	7.9%	4.0%	49.4%	27.9%	10.9%		
